# Supplementary figures and images for: Trust and vaccination intentions: Evidence from Lithuania during the COVID-19 pandemic
Source: PLoS One. 2022 Nov 23;17(11):e0278060. doi: 10.1371/journal.pone.0278060 (PMC9683578; doi:10.1371/journal.pone.0278060)

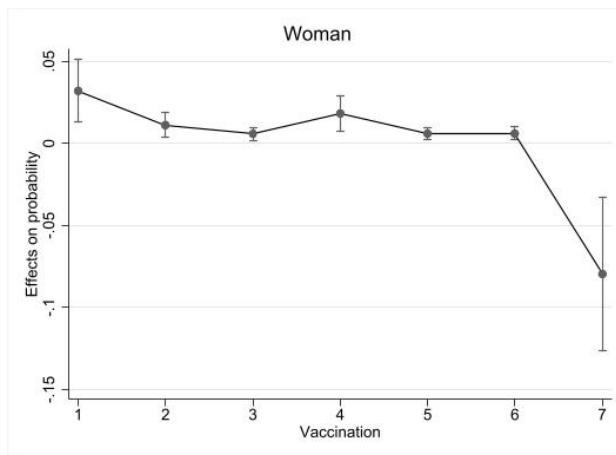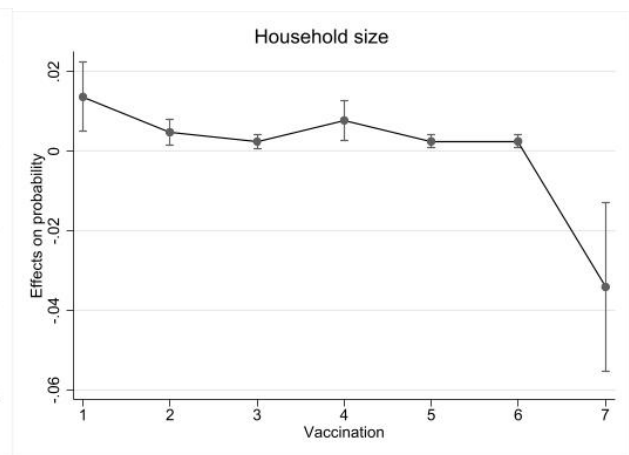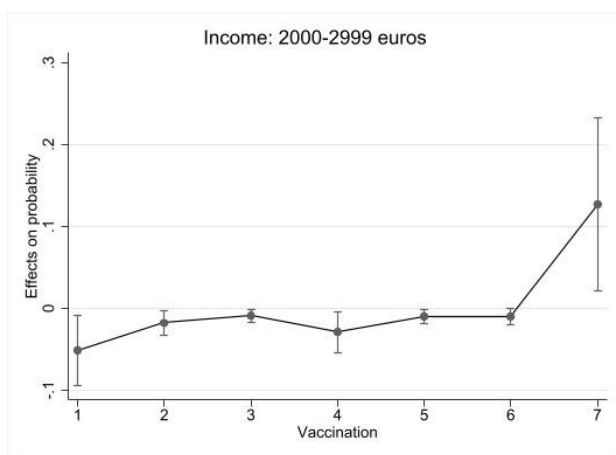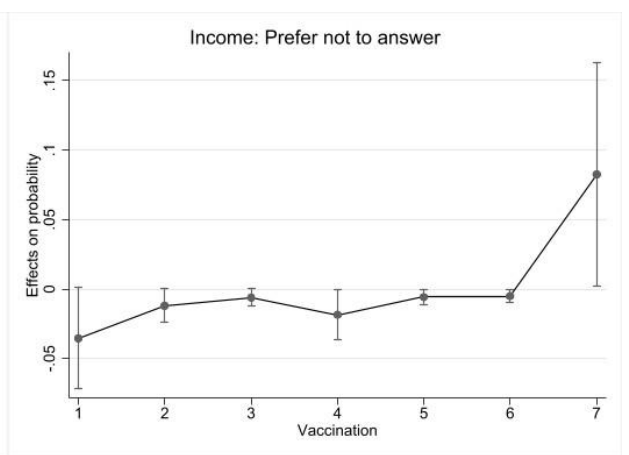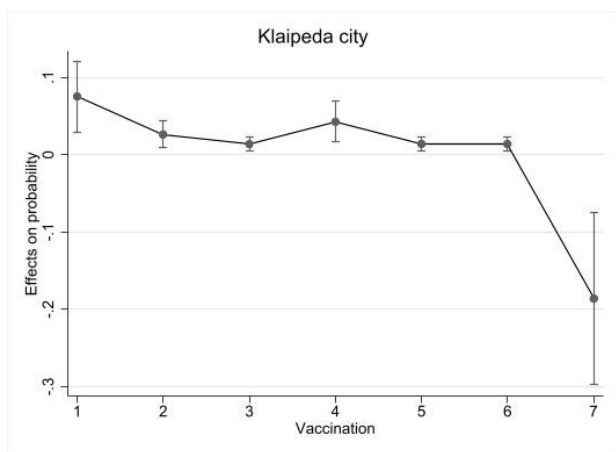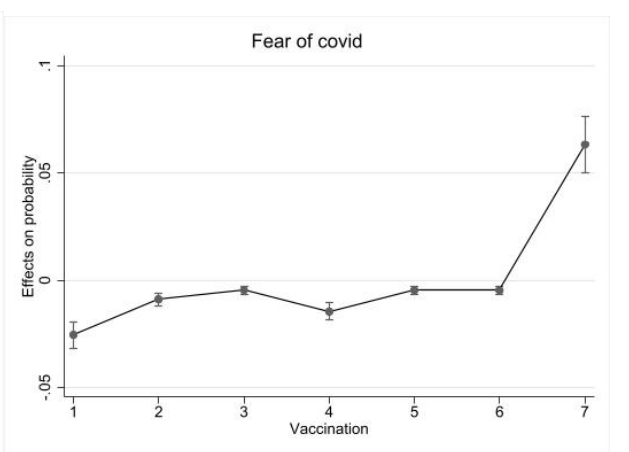

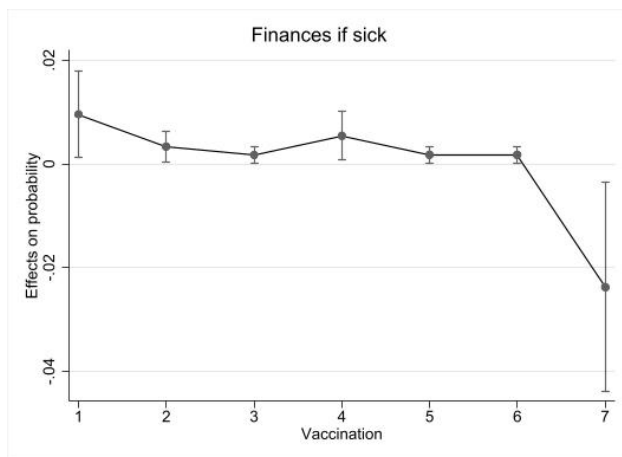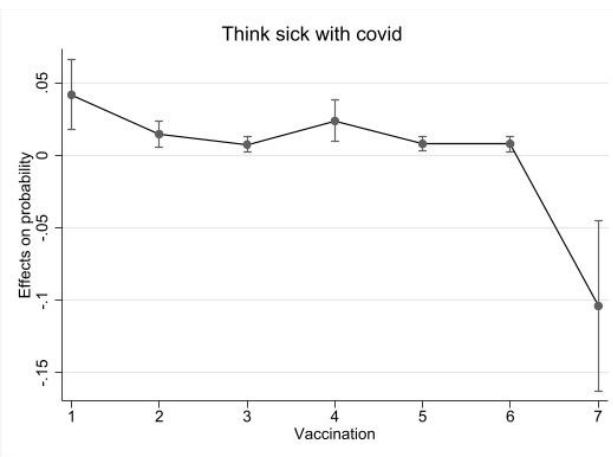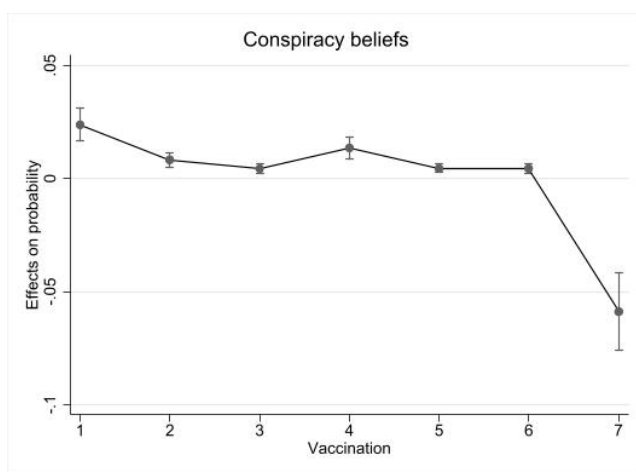

Supplement: S1 Fig — Note: The figures report the estimated average marginal effects of the control variables with 95% confidence intervals from the baseline ordered logistic regression model. The horizontal axis represents answers to the vaccination question: “I will get vaccinated as soon as a free COVID-19 vaccine becomes available to me.” Answers range from 1 = “Strongly disagree” to 7 = “Strongly agree.” We report average marginal effects only of those control variables that have logit coefficients that are statistically significant at least at a 5% significance level. (PDF) [file pone.0278060.s001.pdf]
