# Supplementary material for: Trust and vaccination intentions: Evidence from Lithuania during the COVID-19 pandemic
Source: PLoS One. 2022 Nov 23;17(11):e0278060. doi: 10.1371/journal.pone.0278060 (PMC9683578; doi:10.1371/journal.pone.0278060)
Supplement: S1 Table — (PDF) [file pone.0278060.s002.pdf]

**Variable**	**Definition**
Vaccination	Ordinal variable of agreement to the statement: “I will get vaccinated as soon as a free COVID-19 vaccine becomes available to me.” 1 = “Strongly disagree, 2 = “Disagree,” 3 = “Somewhat disagree,” 4 = “Neither agree nor disagree,” 5 = “Somewhat agree,” 6 = “Agree,” 7 = “Strongly agree.”
Trust in strangers	Continuous variable of answers to the statement: “In general, how much do you trust people you do not know personally?” 1 = “Do not trust at all,” 2 = “Do not trust,” 3 = “Somewhat do not trust,” 4 = “Neither trust nor distrust,” 5 = “Somewhat trust,” 6 = “Trust,” 7 = “Trust completely.”
Trust in government	Continuous variable of answers to the statement: “In general, how much do you trust country’s government authorities?” 1 = “Do not trust at all,” 2 = “Do not trust,” 3 = “Somewhat do not trust,” 4 = “Neither trust nor distrust,” 5 = “Somewhat trust,” 6 = “Trust,” 7 = “Trust completely.”
Trust in healthcare	Continuous variable of answers to the statement: “In general, how much do you trust country’s healthcare system?” 1 = “Do not trust at all,” 2 = “Do not trust,” 3 = “Somewhat do not trust,” 4 = “Neither trust nor distrust,” 5 = “Somewhat trust,” 6 = “Trust,” 7 = “Trust completely.”

Trust in science	Continuous variable of answers to the statement: “In general, how much do you trust science?” 1 = “Do not trust at all,” 2 = “Do not trust,” 3 = “Somewhat do not trust,” 4 = “Neither trust nor distrust,” 5 = “Somewhat trust,” 6 = “Trust,” 7 = “Trust completely.”
Trust in pharma	Continuous variable of answers to the statement: “In general, how much do you trust pharmaceutical companies?” 1 = “Do not trust at all,” 2 = “Do not trust,” 3 = “Somewhat do not trust,” 4 = “Neither trust nor distrust,” 5 = “Somewhat trust,” 6 = “Trust,” 7 = “Trust completely.”
Trust in media	Continuous variable of answers to the statement: “In general, how much do you trust country’s media?” 1 = “Do not trust at all,” 2 = “Do not trust,” 3 = “Somewhat do not trust,” 4 = “Neither trust nor distrust,” 5 = “Somewhat trust,” 6 = “Trust,” 7 = “Trust completely.”
Personal health	Continuous variable of agreement to the statement: “In general, I am physically healthy.” 1 = “Strongly disagree, 2 = “Disagree,” 3 = “Somewhat disagree,” 4 = “Neither agree, nor disagree,” 5 = “Somewhat agree,” 6 = “Agree,” 7 = “Strongly agree.”
Family health	Continuous variable of agreement to the statement: “In general, my closest family members are physically healthy.” 1 = “Strongly disagree, 2 = “Disagree,” 3 = “Somewhat disagree,” 4 = “Neither agree, nor disagree,” 5 = “Somewhat agree,” 6 = “Agree,” 7 = “Strongly agree.”

Diagnosed with covid	Binary variable of answer to the question: "Have you been diagnosed with COVID-19?" 1 = "Yes"; 0 = "No."
Think sick with covid	Binary variable of the answer to the question: "Do you think you have had COVID-19, but have not been diagnosed using a test?" 1 = "Yes"; 0 = "No."
Conspiracy beliefs	Continuous variable of agreement to the statement: "The 5G mobile technology is directly related to the COVID-19 pandemic." 1 = "Strongly disagree, 2 = "Disagree," 3 = "Somewhat disagree," 4 = "Neither agree, nor disagree," 5 = "Somewhat agree," 6 = "Agree," 7 = "Strongly agree."
Risk preferences	Continuous variable of agreement to the statement: "In general, I am willing to take risks." 1 = "Strongly disagree, 2 = "Disagree," 3 = "Somewhat disagree," 4 = "Neither agree, nor disagree," 5 = "Somewhat agree," 6 = "Agree," 7 = "Strongly agree."
Fear of covid	Continuous variable of agreement to the statement: "I fear getting sick with COVID-19." 1 = "Strongly disagree, 2 = "Disagree," 3 = "Somewhat disagree," 4 = "Neither agree, nor disagree," 5 = "Somewhat agree," 6 = "Agree," 7 = "Strongly agree."
Finances if sick	Continuous variable of answer to the question: "How would your financial situation change if the main provider of your family got sick with COVID-19 and could not work for one month?" 1 = "Would deteriorate a lot," 2 = "Would deteriorate," 3 = "Would

	somewhat deteriorate," 4 = "Would neither improve, nor deteriorate," 5 = "Would somewhat improve," 6 = "Would improve," 7 = "Would improve a lot."
Age	Numerical answer to the question: "What is your age?"
Woman	Binary variable of answer to the question: "What is your gender?" 1 = Woman; 0 = Man.
Higher education	Binary variable of answer to the question: "What is your education?" 1 = Higher; 0 = Otherwise.
Employed part-time	Binary variable of answer to the question: "What is your employment status?" 1 = Work part-time; 0 = Otherwise.
Self-employed	Binary variable of answer to the question: "What is your employment status?" 1 = Self-employed; 0 = Otherwise.
Retired	Binary variable of answer to the question: "What is your employment status?" 1 = Retired; 0 = Otherwise.
Student	Binary variable of answer to the question: "What is your employment status?" 1 = Student; 0 = Otherwise.
Unemployed	Binary variable of answer to the question: "What is your employment status?" 1 = Unemployed; 0 = Otherwise.
Other	Binary variable of answer to the question: "What is your employment status?" 1 = Other; 0 = Otherwise.

Household size	Numerical answer to the question: "How many people live in your household?"
Married or live with partner	Binary variable of the answer to the question: "What is your marital status?" 1 = Married or live with partner; 0 = Single or divorced.
No work from home	Binary variable of the answer to the question: "Does your job allow you to work from home?" 1 = No; 0 = Otherwise.
Lithuanian	Binary variable of the answer to the question: "What is your nationality?" 1 = Lithuanian; 0 = Otherwise.
City or town	Binary variable of the answer to the question: "How would you define the size of the settlement you live in?" 1 = City or town; 0 = Rural area
Vilnius city	Binary variable of the answer to the question: "Which municipality do you live in?" 1 = Vilnius City Municipality; 0 = Otherwise.
Kaunas city	Binary variable of the answer to the question: "Which municipality do you live in?" 1 = Kaunas City Municipality; 0 = Otherwise.
Klaipeda city	Binary variable of the answer to the question: "Which municipality do you live in?" 1 = Klaipeda City Municipality; 0 = Otherwise.
500–999 euros	Binary variable of the answer to the question: "What is the aggregate net income of your household in euros (including work-related income, unemployment benefits, sickness benefits, scholarships,

	pensions, and other types of income)?” 1 = 500–999 euros; 0 = Otherwise.
1000—1999 euros	Binary variable of the answer to the question: “What is the aggregate net in- come of your household in euros (including work-related income, unemployment benefits, sickness benefits, scholarships, pensions, and other types of income)?” 1 = 1,000—1,999 euros; 0 = Otherwise.
2000—2999 euros	Binary variable of the answer to the question: “What is the aggregate net in- come of your household in euros (including work-related income, unemployment benefits, sickness benefits, scholarships, pensions, and other types of income)?” 1 = 2,000—2,999 euros; 0 = Otherwise.
>3000 euros	Binary variable of the answer to the question: “What is the aggregate net in- come of your household in euros (including work-related income, unemployment benefits, sickness benefits, scholarships, pensions, and other types of income)?” 1 = More than 3,000 euros; 0 = Otherwise.
Prefer not to answer	Binary variable of the answer to the question: “What is the aggregate net in- come of your household in euros (including work-related income, unemployment benefits, sickness benefits, scholarships, pensions, and other types of income)?” 1 = Prefer not to answer this question; 0 = Otherwise.
