# Supplementary material for: Trust and vaccination intentions: Evidence from Lithuania during the COVID-19 pandemic
Source: PLoS One. 2022 Nov 23;17(11):e0278060. doi: 10.1371/journal.pone.0278060 (PMC9683578; doi:10.1371/journal.pone.0278060)
Supplement: S2 Table — (PDF) [file pone.0278060.s003.pdf]

**Characteristic**	**Frequency (%)**	
	Survey sample	Lithuanian population
Age groups:		
18-29	16.8	17.2
30-39	15.0	15.7
40-49	16.4	16.1
50-59	18.8	18.3
60-69	15.3	15.3
70+	17.7	17.5
Gender:		
Woman	55.4	54.3
Man	44.6	45.7
Size of settlement:		
City or town	67.0	67.3
Rural area	33.0	32.7
District:		
Alytus	4.9	4.9
Kaunas	20.3	20.2
Klaipeda	11.0	11.3
Marijampole	5.1	4.9
Panevezys	8.1	7.7
Siauliai	9.5	9.5
Taurage	3.4	3.3
Telsiai	4.4	4.6
Utena	4.6	4.6
Vilnius	28.7	29.0
Education:		
Higher	70.3	
Other	29.7	
Nationality:		

Lithuanian	92.8
Other	7.2
Employment:	
Employed (full-time)	49.7
Retired	22.7
Unemployed	10.4
Employed (part-time)	4.8
Student	3.8
Self-employed	3.4
Other	5.2
Household size:	
0-1	17.0
2-3	59.4
4-5	22.2
6-7	1.4
Marital status:	
Married or live with a partner	70.9
Single or divorced	29.1
Possibility to work from home:	
Yes	38.1
No	44.7
Somewhat	17.2
Place of residence:	
Vilnius city	18.0
Kaunas city	9.5
Klaipeda city	3.4
Other	69.1
Income:	
0–499 euros	13.5
500–999 euros	27.1

1000—1999 euros	27.2
2000—2999 euros	9.5
>3000 euros	3.5
Prefer not to answer	19.2
In general, how much do you trust people you do not know personally?	
Do not trust at all	11.5
Do not trust	20.0
Do not trust somewhat	12.5
Neither trust, nor distrust	32.7
Trust somewhat	15.8
Trust	6.5
Trust completely	1.0
In general, how much do you trust the country's government authorities?	
Do not trust at all	7.5
Do not trust	12.8
Do not trust somewhat	14.4
Neither trust, nor distrust	17.9
Trust somewhat	25.2
Trust	19.7
Trust completely	2.5
In general, how much do you trust the country's healthcare system?	
Do not trust at all	6.6
Do not trust	10.4
Do not trust somewhat	12.5
Neither trust, nor distrust	15.8
Trust somewhat	25.4
Trust	24.5
Trust completely	4.8
In general, how much do you trust science?	
Do not trust at all	1.0

Do not trust	1.4
Do not trust somewhat	3.0
Neither trust, nor distrust	10.2
Trust somewhat	15.1
Trust	45.6
Trust completely	23.7
In general, how much do you trust pharmaceutical companies?	
Do not trust at all	8.2
Do not trust	9.6
Do not trust somewhat	11.7
Neither trust, nor distrust	22.5
Trust somewhat	23.1
Trust	20.7
Trust completely	4.2
In general, how much do you trust the country's media?	
Do not trust at all	13.6
Do not trust	15.9
Do not trust somewhat	12.1
Neither trust, nor distrust	20.4
Trust somewhat	22.8
Trust	13.6
Trust completely	1.6
In general, I am physically healthy:	
Strongly disagree	1.6
Disagree	5.1
Disagree somewhat	5.9
Neither agree, nor disagree	12.2
Agree somewhat	20.8
Agree	37.5
Strongly agree	16.9

In general, my closest family members are physically healthy:	
Strongly disagree	1.5
Disagree	3.5
Disagree somewhat	8.2
Neither agree, nor disagree	15.9
Agree somewhat	19.5
Agree	37.6
Strongly agree	13.8
Have you been diagnosed with COVID-19?	
Yes	7.3
No	92.7
Do you think you have had COVID-19, but it has not been diagnosed?	
Yes	14.5
No	85.5
How would your financial situation change if the main provider of your family got sick with COVID-19 and could not work for one month?	
Deteriorate a lot	17.8
Deteriorate	21.8
Deteriorate somewhat	25.6
Neither deteriorate, nor improve	33.7
Improve somewhat	0.5
Improve	0.4
Improve a lot	0.2
I fear getting sick with COVID-19:	
Strongly disagree	6.4
Disagree	5.7
Disagree somewhat	4.0
Neither agree, nor disagree	22.0
Agree somewhat	12.5
Agree	23.1
Strongly agree	26.3

In general, I am willing to take risks:	
Strongly disagree	12.7
Disagree	23.2
Disagree somewhat	11.9
Neither agree, nor disagree	25.4
Agree somewhat	15.7
Agree	7.9
Strongly agree	3.2
The 5G mobile technology is directly related to the COVID-19 pandemic:	
Strongly disagree	61.3
Disagree	17.2
Disagree somewhat	2.4
Neither agree, nor disagree	15.8
Agree somewhat	1.2
Agree	1.2
Strongly agree	0.9
