# Supplementary material for: Trust and vaccination intentions: Evidence from Lithuania during the COVID-19 pandemic
Source: PLoS One. 2022 Nov 23;17(11):e0278060. doi: 10.1371/journal.pone.0278060 (PMC9683578; doi:10.1371/journal.pone.0278060)
Supplement: S3 Table — (PDF) [file pone.0278060.s004.pdf]

	N	Mean	Standard deviation
**Dependent variables:**			
Vaccination	973	5.366	2.091
**Independent variables:**			
Trust in strangers	973	3.448	1.473
Trust in government	973	4.095	1.609
Trust in healthcare	973	4.358	1.635
Trust in science	973	5.689	1.200
Trust in pharma	973	4.216	1.622
Trust in media	973	3.702	1.677
**Control variables:**			
Age	973	49.74	16.97
Woman	973	0.554	0.497
Higher education	973	0.703	0.457
Employed full-time	973	0.496	0.500
Employed part-time	973	0.048	0.214
Self-employed	973	0.034	0.181
Retired	973	0.227	0.419
Student	973	0.038	0.191
Unemployed	973	0.104	0.305
Other	973	0.052	0.223
Household size	973	2.601	1.219
Married or live with partner	973	0.709	0.454
No work from home	973	0.447	0.497
Lithuanian	973	0.928	0.259
City or town	973	0.670	0.470
Vilnius city	973	0.180	0.384
Kaunas city	973	0.095	0.293
Klaipeda city	973	0.034	0.181

0–499 euros	973	0.135	0.341
500–999 euros	973	0.271	0.445
1000—1999 euros	973	0.272	0.445
2000—2999 euros	973	0.094	0.293
>3000 euros	973	0.035	0.184
Prefer not to answer	973	0.192	0.394
Personal health	973	5.253	1.439
Family health	973	5.165	1.391
Diagnosed with covid	973	0.073	0.260
Think sick with covid	973	0.145	0.352
Finances if sick	973	2.794	1.147
Fear of covid	973	5.033	1.787
Risk preferences	973	3.448	1.626
Conspiracy beliefs	973	1.860	1.338
