# Supplementary material for: Trust and vaccination intentions: Evidence from Lithuania during the COVID-19 pandemic
Source: PLoS One. 2022 Nov 23;17(11):e0278060. doi: 10.1371/journal.pone.0278060 (PMC9683578; doi:10.1371/journal.pone.0278060)
Supplement: S4 Table — Note: The table shows Spearman’s correlation coefficients. P-values are provided in parentheses. (PDF) [file pone.0278060.s005.pdf]

	Vaccination	*Trust in strangers*	*Trust in government*	*Trust in healthcare*	*Trust in science*	*Trust in pharma*	*Trust in media*
Vaccination	1.0000						
Trust in strangers	0.0730<br>(0.023)	1.0000					
Trust in government	0.4260<br>(<0.001)	0.3301<br>(<0.001)	1.0000				
Trust in healthcare	0.3867<br>(<0.001)	0.2546<br>(<0.001)	0.7523<br>(<0.001)	1.0000			
Trust in science	0.4247<br>(<0.001)	0.1030<br>(<0.001)	0.5021<br>(<0.001)	0.5246<br>(<0.001)	1.0000		
Trust in pharma	0.4090<br>(<0.001)	0.1879<br>(<0.001)	0.6156<br>(<0.001)	0.5610<br>(<0.001)	0.4581<br>(<0.001)	1.0000	
Trust in media	0.3569<br>(<0.001)	0.2347<br>(<0.001)	0.5577<br>(<0.001)	0.4959<br>(<0.001)	0.3522<br>(<0.001)	0.5388<br>(<0.001)	1.0000
