# Supplementary material for: Trust and vaccination intentions: Evidence from Lithuania during the COVID-19 pandemic
Source: PLoS One. 2022 Nov 23;17(11):e0278060. doi: 10.1371/journal.pone.0278060 (PMC9683578; doi:10.1371/journal.pone.0278060)
Supplement: S5 Table — Note: The table reports the logit coefficients obtained by estimating the ordered logit regression model with a single trust variable and no controls. The dependent variable is a 7-category variable vaccination. Standard errors are presented in parentheses below the coefficients. *** p < 0.01, ** p < 0.05, * p < 0.1. (PDF) [file pone.0278060.s006.pdf]

Model	Independent variable	Logit coefficient
**1.1**	*Trust in strangers*	0.090** (0.041)
**1.2**	*Trust in government*	0.576*** (0.042)
**1.3**	*Trust in healthcare*	0.496*** (0.040)
**1.4**	*Trust in science*	0.695*** (0.055)
**1.5**	*Trust in pharma*	0.552*** (0.041)
**1.6**	*Trust in media*	0.435*** (0.039)
