# Supplementary material for: Trust and vaccination intentions: Evidence from Lithuania during the COVID-19 pandemic
Source: PLoS One. 2022 Nov 23;17(11):e0278060. doi: 10.1371/journal.pone.0278060 (PMC9683578; doi:10.1371/journal.pone.0278060)
Supplement: S6 Table — Note: The table reports the logit coefficients of trust variables obtained by estimating the ordered logit regression model with a single trust variable and all controls. We control for respondents’ sociodemographic characteristics, health, conspiracy beliefs, fears of getting sick with COVID-19, impact on finances in the case of COVID-19, and risk preferences. The dependent variable is a 7-category variable vaccination. Standard errors are presented in parentheses below the coefficients. p < 0.01, ** p < 0.05, * p < 0.1. (PDF) [file pone.0278060.s007.pdf]

**Model**	**Independent variable**	**Logit coefficient**
**2.1**	*Trust in strangers*	0.065
		(0.047)
**2.2**	*Trust in government*	0.484***
		(0.047)
**2.3**	*Trust in healthcare*	0.424***
		(0.044)
**2.4**	*Trust in science*	0.526***
		(0.062)
**2.5**	*Trust in pharma*	0.495***
		(0.046)
**2.6**	*Trust in media*	0.313***
		(0.043)
