# Supplementary material for: Trust and vaccination intentions: Evidence from Lithuania during the COVID-19 pandemic
Source: PLoS One. 2022 Nov 23;17(11):e0278060. doi: 10.1371/journal.pone.0278060 (PMC9683578; doi:10.1371/journal.pone.0278060)
Supplement: S7 Table — Note: The table reports the logit coefficients obtained by estimating the ordered logit regression model with all trust variables and all controls (baseline specification). The dependent variable is a 7-category variable vaccination. Standard errors are presented in parentheses below the coefficients. *** p < 0.01, ** p < 0.05, * p < 0.1. (PDF) [file pone.0278060.s008.pdf]

Independent variable	Logit coefficient
Trust in strangers	-0.101**
	(0.051)
Trust in government	0.223***
	(0.074)
Trust in healthcare	0.064
	(0.067)
Trust in science	0.236***
	(0.070)
Trust in pharma	0.284***
	(0.058)
Trust in media	0.032
	(0.052)
**Controls:**	
Age	0.011*
	(0.006)
Woman	-0.483***
	(0.146)
Higher education	0.089
	(0.160)
Employed part-time	0.027
	(0.317)
Self-employed	-0.352
	(0.362)

Retired	0.078
	(0.255)
Student	0.059
	(0.382)
Unemployed	0.138
	(0.230)
Other	-0.615**
	(0.292)
Household size	-0.207***
	(0.066)
Married or live with partner	0.102
	(0.173)
No work from home	-0.167
	(0.144)
Lithuanian	-0.028
	(0.256)
City or town	0.004
	(0.165)
Vilnius city	-0.128
	(0.196)
Kaunas city	-0.397
	(0.242)
Klaipeda city	-1.130***
	(0.347)

500–999 euros	0.259
	(0.227)
1000—1999 euros	0.309
	(0.249)
2000—2999 euros	0.774**
	(0.332)
>3000 euros	0.787*
	(0.432)
Prefer not to answer	0.501**
	(0.252)
Personal health	-0.095
	(0.064)
Family health	0.007
	(0.062)
Diagnosed with covid	0.335
	(0.258)
Think sick with covid	-0.630***
	(0.184)
Finances if sick	-0.144**
	(0.063)
Fear of covid	0.383***
	(0.044)
Risk preferences	0.061
	(0.045)

Conspiracy beliefs	-0.357***
	(0.055)
