# Supplementary material for: Trust and vaccination intentions: Evidence from Lithuania during the COVID-19 pandemic
Source: PLoS One. 2022 Nov 23;17(11):e0278060. doi: 10.1371/journal.pone.0278060 (PMC9683578; doi:10.1371/journal.pone.0278060)
Supplement: S8 Table — Note: The table reports results from Likelihood ratio tests. The tests were conducted to evaluate the difference between nested models, that is, a more restrictive and a less restrictive model. The first column of the table shows which models are compared. LR chi-sq. gives the chi-square statistic for the likelihood ratio test. DF gives the degrees of freedom equal to the difference in the number of degrees of freedom between the two models that are compared. (PDF) [file pone.0278060.s009.pdf]

**Models compared**	**LR chi-sq.**	**DF**	**P-value**
1.1 vs 2.1	367.19	30	<0.001
1.2 vs 2.2	275.45	30	<0.001
1.3 vs 2.3	297.11	30	<0.001
1.4 vs 2.4	267.39	30	<0.001
1.5 vs 2.5	298.56	30	<0.001
1.6 vs 2.6	289.71	30	<0.001
2.1 vs 3	168.24	5	<0.001
2.2 vs 3	60.07	5	<0.001
2.3 vs 3	76.02	5	<0.001
2.4 vs 3	96.13	5	<0.001
2.5 vs 3	47.24	5	<0.001
2.6 vs 3	116.39	5	<0.001
