# Supplementary material for: Trust and vaccination intentions: Evidence from Lithuania during the COVID-19 pandemic
Source: PLoS One. 2022 Nov 23;17(11):e0278060. doi: 10.1371/journal.pone.0278060 (PMC9683578; doi:10.1371/journal.pone.0278060)
Supplement: S9 Table — Note: The table reports results obtained by estimating the baseline specification of our model using the OLS estimator. Variable vaccination is the dependent variable. It is regressed on all trust variables and all controls in a single model. We control for sociodemographic characteristics, health status, experience with COVID-19, conspiracy beliefs, fears of getting sick with COVID-19, impact on finances in the case of getting sick with COVID-19, and risk preferences. Robust standard errors are provided in parentheses *** p < 0.01, ** p < 0.05, * p < 0.1. (PDF) [file pone.0278060.s010.pdf]

| VARIABLES | Coefficients |
|---|---|
| *Trust in strangers* | -0.037 |
| | (0.042) |
| *Trust in government* | 0.130** |
| | (0.064) |
| *Trust in healthcare* | 0.087 |
| | (0.058) |
| *Trust in science* | 0.186*** |
| | (0.064) |
| *Trust in pharma* | 0.244*** |
| | (0.048) |
| *Trust in media* | 0.027 |
| | (0.043) |
| *Personal health* | -0.059 |
| | (0.049) |
| *Family health* | -0.019 |
| | (0.049) |
| *Diagnosed with covid* | 0.202 |
| | (0.204) |
| *Think sick with covid* | -0.527*** |
| | (0.164) |
| *Finances if sick* | -0.116** |
| | (0.051) |
| *Fear of covid* | 0.287*** |
| | (0.039) |
| *Risk preferences* | 0.067* |
| | (0.038) |
| *Conspiracy beliefs* | -0.304*** |
| | (0.051) |
| *Age* | 0.009* |
| | (0.005) |
| *Female* | -0.287*** |
| | (0.110) |
| *Higher education* | 0.041 |
| | (0.127) |
| *Employed part-time* | 0.050 |
| | (0.207) |
| *Self-employed* | -0.243 |
| | (0.283) |
| *Retired* | -0.019 |
| | (0.181) |
| *Student* | 0.006 |
| | (0.328) |
| *Unemployed* | -0.049 |

| | |
|---|---|
| | (0.202) |
| *Other* | -0.528* |
| | (0.275) |
| *Household size* | -0.186*** |
| | (0.059) |
| *Married or live with partner* | 0.130 |
| | (0.142) |
| *No work from home* | -0.244** |
| | (0.112) |
| *Lithuanian* | 0.061 |
| | (0.220) |
| *City or town* | -0.044 |
| | (0.132) |
| *Vilnius city* | 0.003 |
| | (0.142) |
| *Kaunas city* | -0.129 |
| | (0.181) |
| *Klaipeda city* | -0.736** |
| | (0.290) |
| *500-999 euros* | 0.136 |
| | (0.179) |
| *1000-1999 euros* | 0.301 |
| | (0.196) |
| *2000-2999 euros* | 0.512* |
| | (0.274) |
| *>3000 euros* | 0.551 |
| | (0.358) |
| *Prefer not to answer* | 0.418** |
| | (0.210) |
| Constant | 2.030*** |
| | (0.599) |
| Observations | 973 |
| R-squared | 0.443 |
