# Supplementary material for: Trust and vaccination intentions: Evidence from Lithuania during the COVID-19 pandemic
Source: PLoS One. 2022 Nov 23;17(11):e0278060. doi: 10.1371/journal.pone.0278060 (PMC9683578; doi:10.1371/journal.pone.0278060)
Supplement: S1 Text — (PDF) [file pone.0278060.s011.pdf]

Below follows the complete questionnaire. The order of questions provided here follows the original structure of the questionnaire. The questions are translated from Lithuanian.

1. What is your age?

   [Enter a number]

2. What is your gender?

   [Possible answers: woman, man.]

3. How would you define the size of the settlement you live in?

   [Possible answers: city or town, rural area]

4. Which county do you live in?

   [Possible answers: Alytus County, Kaunas County, Klaipeda County, Marijampole County, Panevezys County, Siauliai County, Taurage County, Telsiai County, Utena County, Vilnius County]

5. Which municipality do you live in?

   [Possible answers: Alytus city municipality, Alytus district municipality, Druskininkai municipality, Lazdijai district municipality, Varena district municipality, Birstonas municipality, Jonava district municipality, Kaisiadoriai district municipality, Kaunas city municipality, Kaunas district municipality, Kedainiai district municipality, Prienai district municipality, Raseiniai district municipality, Klaipeda city municipality, Kretinga district municipality, Neringa municipality, Palanga city municipality, Skuodas district municipality, Silute district municipality, Kalvarija municipality, Kazlu Ruda municipality, Marijampole municipality, Sakiai district municipality, Vilkaviskis district municipality, Birzai district municipality, Kupiskis district municipality, Panevezys city municipality, Panevezys district municipality, Pasvalys district municipality, Rokiskis district municipality, Akmene district municipality, Joniskis district municipality, Kelme district municipality, Pakruojis district municipality,

Siauliai city municipality, Siauliai district municipality, Jurbarkas district municipality, Pagegiai municipality, Silale district municipality, Taurage district municipality, Mazeikiai district municipality, Plunge district municipality, Rietavas municipality, Telsiai district municipality, Anyksciai district municipality, Ignalina district municipality, Moletai district municipality, Utena district municipality, Visaginas municipality, Zarasai district municipality, Elektrenai municipality, Salcininkai district municipality, Sirvintos district municipality, Svencioniai district municipality, Trakai district municipality, Ukmerge district municipality, Vilnius city municipality, Vilnius district municipality]

6. To what extent do the following statements describe your behavior last week?
   - I spent my free time only with people whom I live with.
   - I did not travel outside the municipality I live in for personal reasons.
   - I consciously kept a distance from other people in public.
   - In public I wore a mask that covers my mouth and nose.

[Answers on a 7-point scale, ranging from 1 = Does not apply at all to 7 = Applies very much.]

7. To what extent do you agree with the following statements?
   - If I got COVID-19 like symptoms (e.g. loss of taste) tomorrow, I would get tested for COVID-19.
   - If I got COVID-19 like symptoms (e.g. loss of taste) tomorrow, I would isolate myself from society.
   - If I were diagnosed with COVID-19, I would inform the people I had contact with about this.
   - I will get vaccinated as soon as a free COVID-19 vaccine becomes available to me.

- If people who are not vaccinated against COVID-19 were banned from receiving some public services (e.g. attending public events), this would encourage me to get vaccinated.
- If I received more reliable information that the COVID-19 vaccine is safe and effective, this would encourage me to get vaccinated.
- The Lithuanian society is at the moment complying with restrictions that are implemented to manage the COVID-19 pandemic.
- To manage the COVID-19 pandemic in Lithuania, strict restrictions to public life are needed.
- Behavior that gives way to further spread of the coronavirus (e.g. participation in social gatherings), should be punished stricter financially.
- In general, I am physically healthy.
- In general, my closest family members are physically healthy.
- In general, I am willing to take risks.
- I fear getting sick with COVID-19.
- I vote in main elections that are organized in the country (e.g. Lithuanian Parliamentary, Presidential, Municipality elections).
- I pay taxes to the government even when I have the opportunity to avoid them.
- In general, I am a religious person.

[Answers on a 7-point scale, ranging from 1 = Strongly disagree to 7 = Strongly agree.]

8. Do you have ”Korona Stop LT” application installed on your phone, which has a proximity deter- mination function that can warn users about being close to people who are sick with COVID-19?

[Possible answers: Yes; No; No, but I intend to; No, I do not have a smart device; I do not know.]

9. Have you been diagnosed with COVID-19?

   [Possible answers: Yes, No]

10. Do you think you have had COVID-19, but have not been diagnosed with it using a COVID-19 test?

    [Possible answers: Yes, No]

11. How many people, who have been diagnosed with COVID-19, do you know personally?

    [Enter a number]

12. How many people, who have been hospitalized due to COVID-19, do you know personally?

    [Enter a number]

13. In general, how much do you trust:

    - People you do not know personally?
    - The country's government authorities?
    - The country's healthcare system?
    - Science?
    - Pharmaceutical companies?
    - The country's media?

    [Answers on a 7-point scale, ranging from 1 = Do not trust at all to 7 = Trust completely.]

14. How would your financial situation change if the main provider of your family got sick with COVID-19 and could not work for one month?

    [Answers on a 7-point scale, ranging from 1 = Would deteriorate a lot 7 = Would improve a lot.]

15. How did your financial situation change during the COVID-19 pandemic?

    [Answers on a 7-point scale, ranging from 1 = Deteriorated a lot 7 = Improved a lot.]

16. Does your job allow you to work from home?

    [Possible answers: Yes, No, Somewhat]

17. To what extent do you agree with the following statements?

    - The COVID-19 pandemic is a lie.
    - The 5G mobile technology is directly related to the COVID-19 pandemic.
    - Some important global events have been decided by the manipulative actions of a secret influential group of people.

    [Answers on a 7-point scale, ranging from 1 = Strongly disagree to 7 = Strongly agree.]

18. Imagine that your city (or district) municipality is organizing a project. If at least 90% of people from the municipality contributed 10 euros to this project each, the project would be implemented and would yield a benefit that is worth 50 euros to every person living in the municipality (even to those who did not contribute to the project). But if less than 90% contributed, then the project would fail, and the collected money would not be returned. Would you contribute 10 euros to the project?

    [Possible answers: Yes, No]

19. Imagine that you lost a wallet with 200 euros and a driver’s license in it. A person you do not know personally, but who lives near you finds it. How likely is it that he will return the wallet with all your money and everything else inside it to you?

    [Enter a number between 0% (will not return definitely) and 100% (will return definitely).]

20. What is your marital status?

    [Possible answers: Married or live with a partner, Single or divorced]

21. What is your employment status?

    [Possible answers: Employed full-time, Employed part-time, Self-employed, Retired, Student, Unemployed, Other]

22. What is the aggregate net income of your household in euros (including work-related income, unemployment benefits, sickness benefits, scholarships, pensions, and other types of income)?

    [Possible answers: 0–499 euros, 500–999 euros, 1,000—1,999 euros, 2,000—2,999 euros, More than 3,000 euros, Prefer not to answer this question]

23. How many people live in your household?

    [Enter a number]

24. What is your education?

    [Possible answers: Basic, Secondary, Vocational, Higher]

25. What is your nationality?

    [Possible answers: Lithuanian, Polish, Russian, Other]

26. Which political force did you support in the first round of the 2020 Lithuanian parliamentary election?

    [Possible answers: The Centre Party—Nationalists, The Labour Party, The Way of Courage, The Union of Intergenerational Solidarity—Cohesion for Lithuania, The Christian Union, The Freedom Party, The Electoral Action of Poles in Lithuania—Christian Families Alliance, The Lithuanian People's Party, The Liberal Movement of the Republic of Lithuania, The Social Democratic Labour Party of Lithuania, The Social Democratic Party of Lithuania, The Lithuanian Farmers and Greens Union, The Lithuanian Green Party, The National Alliance, Freedom and Justice, Lietuva—VISU,

The Homeland Union—Lithuanian Christian Democrats, Did not vote, Prefer not to answer]
