# Supplementary material for: Trust and vaccination intentions: Evidence from Lithuania during the COVID-19 pandemic
Source: PLoS One. 2022 Nov 23;17(11):e0278060. doi: 10.1371/journal.pone.0278060 (PMC9683578; doi:10.1371/journal.pone.0278060)
Supplement: Attachment — Submitted filename: Response to Reviewers Nov 11 2022.docx [file pone.0278060.s014.docx]

**Response letter for the second round of revisions of**

**“Trust and vaccination intentions: Evidence from Lithuania during the COVID-19 pandemic”**

**(PONE-D-22-13409)**

Dear Professor Gherghina,

Thank you for giving us the opportunity to add additional revisions to our paper “Trust and vaccination intentions: Evidence from Lithuania during the COVID-19 pandemic.” We were encouraged that the reviewers felt positive about the paper. We are grateful for the comments and think the paper improved because of those comments.

Please find below our detailed responses to the points raised by you and the reviewers.

Thank you once again for giving us a chance to revise our paper for a potential publication in *PLOS ONE*.

Best regards,

Laura Galdikiene, Jurate Jaraite, and Agne Kajackaite

**Response to the Academic Editor**

Dear Professor Gherghina ,

Thank you again for giving us the opportunity to revise our paper for a possible publication in *PLOS ONE*.

In the following, we document how we revised the paper in reaction to each of your and reviewers’ comments. Original comments by the review team are written in italics.

1. *Please review your reference list to ensure that it is complete and correct. If you have cited papers that have been retracted, please include the rationale for doing so in the manuscript text, or remove these references and replace them with relevant current references. Any changes to the reference list should be mentioned in the rebuttal letter that accompanies your revised manuscript. If you need to cite a retracted article, indicate the article’s retracted status in the References list and also include a citation and full reference for the retraction notice.*

Thank you for this comment. We have now reviewed our reference list. We found some errors in it which have now been addressed. Some additional references have been added to the list at the request of the reviewers or they were required by the changes introduced in the text of the manuscript. Here is a detailed list of the adjustments we have made to the reference list:

- We have replaced the reference to Galvani et al. (2007) with a reference to Bauch et al. (2003) (see [24] in the manuscript), because we think the latter is more relevant for the statement we are making in the text of the manuscript (see line 107 in the manuscript).
- Bartscher et al. (2021) has been moved down in the reference list (see [32] in the manuscript) due to changes in the text (see lines 121-122 in the manuscript).
- We have added a reference to Šiđanin et al. (2021) (see [47] in the manuscript) to illustrate the relationship between trust in the media and individuals’ attitudes toward vaccinations established in the empirical literature (see lines 165-167 in the manuscript).
- We have adjusted the reference to Saka et al. (2022) to reflect its “accepted for publication” status (see [64] in the manuscript).
- We have removed the reference to Guiso et al. (2008) due to changes in the text (see lines 585-587 in the manuscript).
- We have introduced new references to the reference list (see [66-72] in the manuscript) due to new text added to the manuscript (see line 608-626 in the manuscript). Some of these references (see [66-67, 71] in the manuscript) were recommended by Reviewer 4 (see comments 3 and 4 below).

Thank you for your very helpful comments. We hope we addressed them in a satisfactory way.

**Response to Reviewer 3**

Dear Professor Motta,

Thank you so much for reviewing our paper and for your thoughtful and constructive comments. Revising the paper based on your comments (in italics below) improved the paper significantly.

1. *Line 125: I’m still having a difficult time following the theoretical motivation underlying Hypothesis 1. The authors outline two competing effects regarding how what they call "generalized trust" (more on this in a moment) might be associated with increased vaccine uptake. Notably, they suggest that higher levels of trust could be associated with inaccurate perceptions of pandemic health risks, thereby leading people to engage in *more* risky health behaviors (lines 121-123); including, presumably, not vaccinating. Yet, in line 124, the authors write: "Hence, based on the social capital theory and previous empirical literature..." This link isn’t entirely clear to me. How do we move, conceptually, from a pattern of conflicting results to the sentence quoted above? I would encourage the authors to make additional effort to explicate this conceptual link.*

*Moreover, and I apologize if this is a minor semantic quibble, but I object slightly to the use of the term "generalized trust" -- which is typically used to refer to trust across many different institutional and interpersonal actors (e.g., see: https://onlinelibrary.wiley.com/doi/full/10.1111/ajps.12234) -- as a synonym for trust in people one does not know ("strangers"). I’d encourage the authors to use consistent terminology throughout the piece, perhaps eschewing references to "generalized trust" in this domain.*

Thank you so much for this useful comment. We agree that the conceptual link between generalized trust and vaccinations lacked clarity. We have now explained it in the manuscript. In particular, on pages 6-7 of the manuscript we write the following:

“The social capital theory suggests that social capital, that is, certain shared values, norms, bonds, and trust among people, can help societies overcome the free-rider problem and facilitate cooperation in the pursuit of socially valuable activities [5-6, 25-27]. When it comes to general trust in other people, that is, generalized trust, and, particularly, trust in strangers, more trusting individuals are more willing to cooperate and contribute to the public good, because they view other people as trustworthy and do not think they will be cheated [5]. In the context of vaccinations, this could mean that if people trust others, they are more willing to get vaccinated, because they do not think that others will free-ride and refuse vaccinations needed to stop the spread of the virus.

But empirical evidence on the role of social capital in shaping health behavior is somewhat conflicting. Some authors show that social capital (where trust is an important component) is associated with increased voluntary compliance to non-pharmaceutical interventions, including social distancing during the COVID-19 pandemic [28-31], as well as improved health outcomes, that is, fewer COVID-19 cases and fewer excess deaths per capita [32]. Some empirical studies also find a positive association between generalized trust and vaccination willingness [33-35]. However, some studies on health behavior find the opposite and do not confirm the theoretical considerations of the social capital theory. For example, Jennings et al. [36] show evidence of no significant relationship between generalized trust and vaccination intentions during the COVID-19 pandemic. Deopa and Fortunato [37] and Doganoglu and Ozdenoren [38] find a negative effect of generalized trust on social distancing behavior during the COVID-19 pandemic, arguing that when people trust others, they may believe that other people are sticking to restrictions and feel that it is safe to go out. However, despite this mixed empirical evidence, we base our prediction on the social capital theory. Hence, we hypothesize that:

Hypothesis 1: Trust in strangers predicts higher vaccination intentions (H1).”

Also, thank you for your comment on generalized trust. Many authors define generalized trust as trust in other people in general (Uslaner, 2002; Guiso et al., 2011; Carl and Billari, 2014; Delhey et al., 2011). Generalized trust is most often measured using the question “Generally speaking would you say that most people can be trusted or that you can’t be too careful in dealing with people? (Rosemberg, 1956).” Many scholars also view trust in people one does not know, that is, trust in strangers, as the cornerstone of generalized trust, and argue that generally trusting means that we trust strangers (Uslaner, 2002). Thus, we think that the terms “generalized trust” and “trust in strangers” can be used as synonyms in the manuscript, because they are very closely related concepts, or some would even argue that “trust in strangers” can be referred to as “generalized trust”, at least in a narrow sense. To the best of our knowledge, generalized trust rarely includes trust across different institutional actors. It also distinguishes from particularized trust, that is trust toward familiar others, including friends, neighbors, and co-workers. Since references to “generalized trust” are essential for our review of the related literature, including the theoretical background, and the use of such references are in line with the existing literature, we are inclined to keep them in the manuscript.

However, to ensure that our reasoning is clear, we introduce some minor changes to the text of the manuscript. In the subsection “Present research” on page 9 of the revised manuscript we write: “For interpersonal trust, we explore trust in strangers, because it should help societies overcome the free-riding problem and encourage cooperation between strangers [5-6]. In this paper we focus on trust in strangers instead of the broader definition of generalized trust. The broader definition of generalized trust measures trust in other people in general, which is most often elicited by asking “Generally speaking would you say that most people can be trusted or that you can’t be too careful in dealing with people? [49]”. Generalized trust and trust in strangers are very closely related concepts and are often used as synonyms [5]. However, we think that trust in strangers eliminates the ambiguity inherent in the broader concept of generalized trust.”

2. *Line 165: The authors claim that this survey is nationally representative of the Lithuanian adult population. While I appreciated the authors’ willingness to offer a new table in the Supplementary Materials denoting the basic descriptive properties of their sample, it was not clear to me how these demographics compared to those of the Lithuanian population. I would encourage the authors to therefore amend this supplemental table by including a comparison of sample properties to nationally representative benchmarks. Relatedly, I think the authors could say much more about the process by which the third party survey firm with whom they contracted not only invited individuals to participate in the study (which the authors addressed -- quite well, in my view! -- in the previous round of review) but how these individuals were *sampled.* The authors later note that the firm was taking random draws from an online opt in panel service (line 197). In order for the study to be nationally representative, this would imply that the online opt in sampling frame *itself* is nationally representative; something which is very atypical in online survey research (although I confess that I am not familiar with how this particular firm operates!). Correspondingly, I’d encourage the authors to provide additional information about how their sampling protocols ensure national representativeness.*

Thank you so much for this comment. We have now included a comparison of sample properties to nationally representative benchmarks for main demographic characteristics in the S2 Table. We have also provided some additional information on the sampling process that was made available to us by the company “Norstat” that implemented the survey. In subsection “Survey” on pages 9-10 of the revised manuscript we write the following:

“We employed a data set from a representative incentivized online panel survey conducted on 13–20 January 2021. We hired the company “Norstat” to implement the survey using its online access panel, that is, a group of registered internet users who have agreed to take part in various surveys. For participating in surveys, “Norstat” panel members are rewarded with virtual coins that could be exchanged into gift cards, coupons, or donated to a charity. The database of “Norstat” panel members was collected by the company by conducting member recruitment campaigns and representative surveys of the general population. The company sends individual invitations to potential panel members asking them to join the panel and individuals can then either accept or reject the invitations. The invitation-based system allows the company to ensure a diverse pool of individuals available for nationally representative surveys. Our survey participants were selected from the panel randomly according to the representativeness parameters, including age groups, gender, districts, and size of settlement (urban or rural). Invitations to participate in our survey were sent to potential participants by an automated system via email, which included a link to our questionnaire. The process of sending invitations continued until all sampling quotas for the target groups were fulfilled. The sampling quotas were set according to the population distribution data provided by Statistics Lithuania.”

3. *Tables 1-3: While I appreciate the authors’ willingness to calculate and present more-substantively tractable quantities from their ordered logistic regression models, the piece provides little information (at least, in my reading) of whether or not each independent variable in the model had a statistically significant effect on vaccine uptake. I raise this point because, in theory, statistically insignificant independent variables (e.g., one of the dimensions of trust) could potentially have non-linear and significant marginal effects at some points on the ordered Likert scale on vaccination intentions, while nevertheless not resulting in appreciable change in vaccination intentions that are appreciable from zero. For that reason, some scholars caution against calculating marginal effects and predicted probabilities from statistically insignificant regressors. I would therefore encourage the authors to add information (either to the manuscript’s main text, or to the Tables) denoting whether or not each of the measures of trust yielded a statistically significant increase in vaccination intentions in the ordered logistic regression models, in addition to presenting the marginal effects listed in each table. Alternatively, the authors might consider migrating Table S6 from the supplement to the main text. I completely defer to the authors on this score, regarding which course of action might be best. As long as they make some effort to introduce this information in the main text, I’ll be satisfied!*

Thank you for this useful comment. We have now added several tables with additional results from our estimated regression model as Supporting Information (see S5-S7 Table). These tables report the estimated logit coefficients of trust variables obtained by estimating the baseline model (see S7 Table) as well as the estimated logit coefficients from the reduced form ordered logistic regression models (see S5 and S6 Table). In the subsection “Main results” we have now provided information on the logit coefficients of the trust variables as well as the references to S5-S7 Table. In particular, on pages 17-23 of the revised manuscript we write the following:

“First, we present the results from the simplest regression specification, which regresses *vaccination* on every trust variable in separate regressions without the control variables. As expected, we find the positive association between all trust variables and vaccination intentions. The logit coefficients are statistically significant at least at a 5% significance level (see S5 Table). The average marginal effects of all trust variables are also statistically significant at least at a 5% significance level for all categories of responses to the vaccination question (see columns 1.1-1.6 in Table 1). The average marginal effects of institutional trust variables are larger in numerical terms than those of trust in strangers. Overall, the average marginal effects of trust in strangers are relatively small in numerical terms. On average, an increase in trust in strangers by 1 point is associated with a 2.2%-point greater probability of “agreeing strongly” to getting vaccinated. For trust in the government, the healthcare system, the pharmaceutical companies and the media, an increase in trust by 1 point is associated with around a 10-12%-point greater probability of reporting the highest vaccination intentions. Trust in science demonstrates the largest average marginal effects observed for the highest vaccination intentions (see column 1.4 in Table 1).

Next, we present the results from the regression models that regress vaccination intentions on every of the six trust variables separately and all control variables (sociodemographic characteristics of respondents, their health, conspiracy beliefs, fears of getting sick with COVID-19, impact on finances in the case of COVID-19, and risk preferences). We find that when we control for potential covariates, the logit coefficient of trust in strangers becomes statistically insignificant at any conventional significance level (see S6 Table). This is also reflected in the average marginal effects of trust in strangers, which are also statistically insignificant for all categories of responses to the vaccination question (see column 2.1 in Table 2). But the logit coefficients of the remaining trust variables, that is, trust in the government, trust in healthcare, trust in science, trust in pharma, and trust in the media, remain statistically significant at least at the 1% significance level (see S6 Table). The average marginal effects of these trust variables also keep their signs and remain statistically significant at the 1% level for all categories of vaccination variables (see columns 2.2-2.6 in Table 2). The association between vaccination intentions and the institutional trust variables remains positive. On average, an increase in trust in different institutions involved in the vaccination process by 1 point is associated with a 7.4-9.4%-point greater probability of agreeing strongly to getting vaccinated. Regarding the media, as trust in it goes up by 1 point, the probability of reporting highest vaccination intentions increases by 5.6%-points.

However, the above-reported results from the first two regression models that include each trust variable separately could be misleading. That is, the estimated marginal effects of the trust variables may be biased upward, as the individually included trust variables might be capturing the effects of other trust variables that are omitted from the model. For this reason, the third regression specification, which includes all trust variables and controls, is estimated. This is our baseline model.

Estimating the baseline model yields logit coefficients that are statistically significant at the 1% significance level for trust in the government, trust in science, and trust in pharmaceutical companies (see S7 Table). The average marginal effects of these trust variables also remain statistically significant at the 1% level for all categories of vaccination variables. As trust in these institutions rises, the probability that the individuals report higher vaccination intentions increases (see Table 3). On average, an increase in trust in the government, science, and pharmaceutical companies by 1 point is associated with a 3.7, 3.9, and 4.7%-point greater probability of reporting the highest vaccination intentions, that is, “agreeing strongly” to getting vaccinated, respectively. In other words, higher trust in these institutions reduces the probability of disagreeing, being undecided, and agreeing less than strongly to getting vaccinated. These findings are in line with previous results in the literature [9-10, 33, 43] and provide evidence in favor of hypotheses H2, H4, and H5.

Although some authors found that trust in healthcare [34, 45] and trust in the media [8, 46-48] were associated with higher vaccination intentions, in our case, these effects are potentially reduced by the inclusion of other trust variables, in particular, trust in the government and trust in science. The estimates of our baseline model show that the logit coefficients of trust in healthcare (H3) and trust in the media (H6) are statistically insignificant at any conventional level (see S7 Table). The average marginal effects of these trust variables are also statistically insignificant for all categories of responses to the vaccination question (see Table 3).

Furthermore, we find that when we estimate the baseline model, the logit coefficient of trust in strangers becomes statistically significant at the 5% significance level (see S7 Table). We also find that higher trust in strangers is associated with a lower probability of having high vaccination intentions (see the second column in Table 3). Thus, we do not find evidence in favor of H1. This result contrasts with the findings of some authors [33-35], who show that generalized trust is positively associated with the willingness to get vaccinated. One of the potential explanations of our result could be related to the fact that in this paper we focus on trust in strangers, which is a somewhat different concept than the broader concept of generalized trust analyzed in most other similar studies. Differences in the timing of surveys could also play a role—our survey was conducted quite early in the vaccination process, possibly before most people had internalized the social benefits of vaccinations. Another potential explanation for the negative association between trust in strangers and vaccinations is that when people trust others, they may believe that other people will protect against the disease, for example, by adhering to specialists’ recommendations about safe health behavior during the pandemic and/ or by getting vaccinated, thus, they may feel safer about not rushing to get their vaccine. A few previous studies analyzing the role of generalized trust in explaining social distancing behavior during the COVID-19 pandemic have also found similar results [37-38]. However, our estimated average marginal effects of trust in strangers are relatively small in numerical terms and are statistically significant at the 5% significance level only for the highest and lowest vaccination intentions (see the second column in Table 3), meaning that one should be cautious in drawing strong conclusions from this finding.”

In addition, in S7 Table we have included the logit coefficients of the control variables obtained by estimating our baseline model. We have also included a reference to this table in the manuscript. In subsection “Additional results” on page 24 of the manuscript we write: “In this subsection, we report and discuss the additional findings obtained by estimating the baseline ordered logistic regression model. The control variables that we include in this model provide interesting insights about how individual characteristics, beliefs, and attitudes are associated with vaccination intentions. In S7 Table we report the logit coefficients of all control variables that are included in our baseline model. In S1 Fig we plot the average marginal effects of those control variables that have statistically significant (at least at a 5% significance level) logit coefficients.”

Also, to ensure that we describe all findings obtained by estimating our baseline model that are reported in S7 Table, we have oulined a few additional observations about the additional results to the text of the revised manuscript. In particular, in subsection “Additional results” on pages 24-25 we write: “In addition, individuals who report having higher income as well as those who prefer not to answer the question about their income are more likely to have high vaccination intentions. Some authors have also found a positive association between income and vaccinations [9, 57]. We also find that individuals from Klaipeda—the third largest Lithuanian city and a major seaport with a relatively large Russian-speaking population—are less likely to be in favor of getting vaccinated.” S1 Fig was also complemented with additional graphs plotting the average marginal effects for variables *2000-2999 euros*, *Prefer not to answer*, and *Klaipeda city*.

Thank you again for your very helpful comments. We hope we addressed them in a satisfactory way. And thank you so much for believing that this paper should be accepted after minor revisions. Your comments have encouraged us.

**Response to Reviewer 4**

Dear Reviewer,

Thank you so much for reviewing our paper and for your thoughtful and constructive comments. Revising the paper based on your comments (in italics below) improved the paper significantly.

1. *It seems a bit contradictory to frame the "types" of trusted institutions that correlate with vaccination intention as ones "that are directly related to the development, approval, and distribution of vaccines" (abstract). I mean this is true, but healthcare as an institution certainly falls under this umbrella category as well. How do we reconcile this?*

Thank you so much for this comment. We have now removed references to the category of institutions that are “directly related to the development, approval, and distribution of vaccines.” In particular, in the abstract on page 2 of the revised manuscript we write: “Using unique survey data from Lithuania during the COVID-19 pandemic, we show that trust in government authorities, science, and pharmaceutical companies are important predictors of individual vaccination intentions.”. In the “Conclusions” section we write: “Our survey data show that the intent to get vaccinated is positively associated with trust in the government, science, and pharmaceutical companies.”

2. *There seems to be another contradiction when motivating the case context vis-a-vis the descriptive results. On page 4, the manuscript argues that Lithuania offers an interesting case in part because "Lithuania has suffered greatly from the COVID-19 pandemic and has faced high vaccine skepticism (see subsection “COVID-19 and vaccinations in Lithuania". At the same time, the authors note "Most of respondents expressed a willingness to get vaccinated against COVID-19 (see Fig 1). In total, around 69% of respondents said that they strongly agree, agree, or agree somewhat to receive a vaccine as soon as it becomes available. How do we reconcile that vaccination skepticism is high yet at the same time most people say they want the vaccine? Is it because of a temporal dynamic from time of study to actual vaccinations? Is it because the panel is not really representative of the population (i.e., vaccine-willing individuals are more likely to self-select into panel)? Or is it because initial vaccine willingness is prone to a social desirability factor?*

Thank you for this valuable comment. We agree that our writing in motivating the case context was not entirely accurate. When we write about high vaccine skepticism in the “Novelty of the study” subsection we mean vaccine skepticism in over-50-year-olds and slow vaccination uptake, and when we describe our survey data we talk about vaccine willingness in over-18-year-olds. We are sorry for our imprecision and are grateful for you noting it. We have now adjusted the text of the manuscript. In subsection “Novelty of the study” on page 4, we now write: “Lithuania has suffered greatly from the COVID-19 pandemic and it has faced sluggish vaccinations and high vaccine skepticism among the older population (see subsection “*COVID-19 and vaccinations in Lithuania*”).”

Also, as you correctly noted, there could be a gap between early vaccination intentions, that we analyze in our paper, and actual vaccinations due to temporal dynamics and/ or social desirability bias. However, there is no way to check for this in our analysis, since individual data for vaccine uptake is not publicly available. But we outline this concern as one of the limitations of our study in the “Conclusions” section. In particular, on pages 27-28 we write: “However, there could be other factors than the social desirability bias that could lead to differences between vaccination intentions and the actual vaccine uptake, such as the temporal dynamics. Although in our study, many respondents expressed willingness to get vaccinated, which, according to Jensen et al. [71], should predict a high uptake of COVID-19 vaccines, the observed vaccination process was relatively slow in Lithuania. It may be the case that some individuals had second thoughts about getting vaccinated when the time to receive their vaccine came. To address the social desirability bias as well as other concerns related to the potential gap between vaccine willingness and actual vaccine uptake, future studies could use experimental measures of trust [70, 72] and actual vaccination behavior.”

3. *The latter option takes me to the biggest concern with the study results. How do we know these results are confounded by a latent social desirability factor? People who like to conform to social norms might indicate that they are more trusting (because it is considered the right thing to be) and express greater willingness to get vaccinated (because it is touted as the socially and morally right thing to do)? In other words might social desirability bias be an omitted variable bias in this study? One way to potentially address this somewhat is to draw on studies that have examined the extent to which Covid-19 mitigation behaviors are susceptible to social desirability (e.g., Jensen 2020 and Larsen et al. 2020).*

Thank you so much for this useful comment and the references. We agree that social desirability factor could bias the results. To ensure that we emphasize this limitation enough we have added additional information to the “Conclusions” section on pages 27-28 of the manuscript. We write:

“Second, the study investigates self-reported trust and vaccination intentions, which may suffer from the social desirability bias and be prone to other concerns. The social desirability bias implies that survey respondents may overreport socially desirable and/ or underreport socially undesirable behavior. In our case, to be viewed favorably by others, respondents may indicate that they are more trusting and/ or more willing to get vaccinated than they actually are. But it is likely that this bias is not a very big concern here, as several studies have shown that the social desirability bias for self-reported health behaviors during the COVID pandemic, such as social distancing, is either very small [66] or even non-existent [67]. In addition, some studies have found that self-reported interpersonal and institutional trust predicts experimental trust measures [68-70]. However, there could be other factors than the social desirability bias that could lead to differences between vaccination intentions and the actual vaccine uptake, such as the temporal dynamics. Although in our study, many respondents expressed willingness to get vaccinated, which, according to Jensen et al. [71], should predict a high uptake of COVID-19 vaccines, the observed vaccination process was relatively slow in Lithuania. It may be the case that some individuals had second thoughts about getting vaccinated when the time to receive their vaccine came. To address the social desirability bias as well as other concerns related to the potential gap between vaccine willingness and actual vaccine uptake, future studies could use experimental measures of trust [70, 72] and actual vaccination behavior.”

4. *Finally it can offer additional credence to validity of the self-reported measure of vaccine willingness to demonstrate how it maps onto actual vaccination behavior. Several recent studies show that early willingness strongly predict actual uptake, see for example Jensen, Ayers and Koskan (2022) in PLoS ONE.*

Thank you so much for this comment and the suggested reference. On page 28 of the manuscript we have now added: “Although in our study, many respondents expressed willingness to get vaccinated, which, according to Jensen et al. [71], should predict a high uptake of COVID-19 vaccines, the observed vaccination process was relatively slow in Lithuania.”

Thank you for your very helpful comments. We hope we addressed them in a satisfactory way.

**Additional references:**

1. Carl, N. and Billari, F.C., 2014. Generalized trust and intelligence in the United States. PloS one, 9(3), p.e91786.
2. Delhey, J., Newton, K. and Welzel, C., 2011. How general is trust in “most people”? Solving the radius of trust problem. American Sociological Review, 76(5), pp.786-807.
3. Guiso, L., Sapienza, P. and Zingales, L., 2011. Civic capital as the missing link. Handbook of social economics, 1, pp.417-480.
4. Rosenberg, M., 1956. Misanthropy and political ideology. American sociological review, 21(6), pp.690-695.
5. Uslaner, E. M., 2002. The moral foundations of trust. Cambridge University Press.
